# The Effect of Usual Source of Care on the Association of Annual Healthcare Expenditure with Patients’ Age and Chronic Disease Duration

**DOI:** 10.3390/ijerph15091844

**Published:** 2018-08-27

**Authors:** Sungje Moon, Mankyu Choi

**Affiliations:** 1 Department of Public Health Sciences, Graduate School of Korea University, Seoul 02841, Korea; msjess0329@hotmail.com; 2 BK21PLUS Program in Embodiment: Health-Society Interaction, Korea University, Seoul 02841, Korea

**Keywords:** chronic disease, healthcare expenditure, usual source of care

## Abstract

Along with rapid population aging, the importance of chronic disease management increases with high growth of national healthcare expenditures, and efficient spending on healthcare is required to reduce unnecessary utilizations. For that reason, this study examined the association of annual healthcare expenditure with age and disease duration of chronic patients. Furthermore, the study investigated the effect of usual source of care (USOC) to suggest directions for preventive management of chronic disease. Using Korean Health Panel Study data, this study selected 1481 outpatients, who had out-of-pocket costs for hypertension or diabetes, and their total healthcare and chronic disease management (CDM) costs were examined. With patient aging, CDM cost decreased while the total healthcare cost increased, but longer duration of hypertension or diabetes resulted in increases in both CDM and total healthcare costs. In addition, the moderating effect of USOC indicated that elderly patients had increased CDM costs when they had a regular site for healthcare. In contrast, patients with longer duration had reductions in both CDM and total healthcare costs while having a regular doctor increased CDM cost. The results of this study could be an evidence for future policies to suggest proper preventive management plans for specific subjects.

## 1. Introduction

Rapid worldwide increases in population aging and increased life expectancy are causing high national health expenditures, which increase the importance of the national healthcare system. Concurrently, healthcare systems, which have usually focused on treatment and management of acute diseases, are changing their focus to prevention-centered health care systems for chronic and degenerative diseases [1]. According to World Health Organization (WHO), premature mortality from non-communicable diseases, also known as chronic diseases, accounted for 52% of global deaths under the age of 70 years in 2012 [2]. This indicates that there is a need for prevention and management of the expansion and severity of chronic diseases. Furthermore, these may cause negative health outcomes such as aggravation of complex diseases and increases in national healthcare expenditures.

Chronic disease is usually defined as a disease that lasts for more than a year and requires ongoing or continuous management, and is regarded as preventable even though difficult to cure spontaneously [3,4]. WHO reported that chronic diseases are classified into four major categories: cardiovascular disease, cancer, chronic respiratory disease, and diabetes [2]. However, it is still necessary to standardize the list of chronic diseases because of the wide range of definitions and diagnostic classification systems. For this reason, the Office of the Assistant Secretary of Health (OASH) in the United States also complied the OASH list of 20 chronic diseases [5]. According to a study, which applied the OASH list in South Korea, prevalence of chronic disease was highest in the order of hypertension (16.6%), arthritis (14.4%), diabetes (6.4%) and hyperlipidemia (6.1%); additionally, a higher percentage of out-of-pocket (OOP) costs were covered for these diseases [6]. Since chronic diseases are more likely to involve long-term and sustained medical expenses, there is a greater need to reduce their incidence through preventive management rather than focusing on their treatment. Therefore, to buffer the increasing national healthcare expenditures following from the growth of the elderly population, customized prevention or health promotion plans should be prepared that consider the characteristics of patients who have chronic diseases.

In addition, the WHO has reported that chronic diseases are caused by a number of complex factors, such as genetic, physiological, environmental, and behavioral factors [2]. However, it is also necessary to concentrate on factors, which are changed over time, to consider the characteristics of chronic diseases. As age increases, the prevalence of chronic disease and probability of transition to multiple chronic diseases increases [7]. This means that focusing on the elderly population is required to propose appropriate prevention measures for chronic diseases. However, even though healthcare expenditures for the elderly are substantially greater because of health deterioration and more healthcare utilizations, the primary care costs for disease prevention and management decrease as the age of patients increases [8]. Additionally, factors such as more benign perception of illness and greater perceived illness burden among elderly people with hypertension were found to reduce medication compliance with negative treatment belief [9]. For this reason, it would be required to increase healthcare costs to prevent complications caused by chronic diseases, even though it might be ideal to reduce elderly healthcare expenditure through long-term health promotion. In addition, it is important to consider not only the age of the patients but also the disease duration. A study of vascular complications and death in diabetic patients found that age and duration of disease have independent and different effects, especially in younger patients [10]. Consequently, chronic disease prevention and management should not only consider the patients’ age but also long-term continuity of care depending on disease duration.

Due to increases in chronic diseases and healthcare expenditures, there have been many national projects for prevention and management around the world, and this includes South Korea. By implementing a “clinic-based chronic disease management system”, effective and sustainable chronic disease management has been established with the activation of primary healthcare institutions. Additionally, through the “Hypertension and Diabetes Registration and Management Project”, South Korea has aimed to delay the onset of chronic disease and health deterioration with improving continuity of care and better health behaviors [11]. This national level of management is also intended to reduce the socio-economic burden of chronic diseases. However, the Korea Centers for Disease Control and Prevention confirmed that the rate of patients who controlled hypertension through treatment was 44% even though the diagnosis rate was 65.9%. For diabetes, likewise, the control rate was 26.8%, which was significantly lower than the diagnosis rate of 68.3% [12]. These statistics reflect, in part, that hypertension and diabetes are insufficiently managed after being diagnosed, even though active medicine therapy can prevent the occurrence of serious complications. Continuity of care for chronic diseases, which indicates regularity of visiting a primary care doctor, may increase healthcare expenditure in the short-term by increasing direct medical costs with spending more on prescription drugs [13]. In the long-term, however, continuity of care may reduce the incidence of complications and also reduce overall healthcare expenditures.

Therefore, this study examined usual source of care as a measurement of continuity, and confirmed its moderating effect on healthcare expenditure according to chronic patients’ age and disease duration. Furthermore, this study aimed to suggest a direction for prevention and management plans by predicting healthcare utilization behaviors of chronic patients through their annual healthcare spending, and to reduce the burden of chronic diseases.

## 2. Materials and Methods

### 2.1. Data and Study Design

This study was performed to investigate the effects of patients’ age and duration of chronic diseases on annual medical expenditures using data from the Korea Health Panel Study (KHPS). The KHPS is regarded as providing nationally representative and stratified random sampling data, which has been tracked for 8 years to confirm healthcare utilization and the level of medical expenditure in South Korea. Using this data, the study aims to identify not only the effects of age and duration of chronic disease but also the moderating effect of continuity of care on annual medical expenditures to propose an intervention plan to make medical expenses more efficient. The 5–6th KHPS data (2012–2013) were used to utilize the usual source of care, which was measured only for two years as a parameter of continuity of care.

### 2.2. Study Population

In this study, outpatients who had healthcare expenditures because of hypertension or diabetes were selected as the study population. Based on the Korean Standard Classification of Disease and Cause of Death (KD-6), outpatients diagnosed with hypertension (I10), diabetes (E11-14), or both disease were selected for the study sample. Accordingly, a total of 1481 outpatients were selected for the final sample, excluding those who did not spend OOP costs even when there was medical utilization, and those who did not answer about the costs of medical services and prescription drugs.

### 2.3. Variables

#### 2.3.1. Age and Duration of Diseases

The independent variables for this study were selected as patients’ age and the duration of chronic disease. The questionnaires surveyed the year of birth and the first year that a disease was diagnosed by the doctor, and these were reconstructed as number of years as of 2012. Each patients’ age was confirmed to be over twenty in 2012, and the duration of chronic disease was calculated by the year when hypertension or diabetes was first diagnosed. Having both chronic diseases was determined from the base year of shifting to multiple chronic diseases.

#### 2.3.2. Healthcare Expenditure

Annual healthcare expenditures were specified with OOP costs that arose for outpatients who spent on medical services and prescription drugs in 2012 and 2013. This study classified the annual healthcare expenditure into two variables defined as total healthcare cost and chronic disease management (CDM) cost. Total healthcare cost comprised the entire healthcare expenditure of outpatients who had hypertension, diabetes, or both, and was measured as annual healthcare cost caused by all of the diseases including hypertension and diabetes. However, CDM costs only comprised OOP costs incurred by visits for hypertension and diabetes as major diseases. In addition, an analysis of each cost was conducted with subcategories: medical service, prescription drug, and the total.

#### 2.3.3. Usual Source of Care

The moderating variable of this study was usual source of care (USOC) which was measured whether chronic disease patients possessed it during 2012 and 2013. The questionnaires were composed of whether patients had a particular source of care, such as a regular site or regular doctor, when they were in need of medical advice or became diseased. This study used these variables to confirm the moderating effect on annual healthcare expenditures by measuring continuity of care for hypertension and diabetes through consistent visiting and management by having USOC.

#### 2.3.4. Control Variables

Socioeconomic characteristics were used as control variables, and included sex, marital status, household income, and disability. Furthermore, healthcare utilization of patients was considered with types of medical institutions and healthcare coverage. The types of medical institutions were categorized into four types: general hospitals, hospitals, clinics, and public health centers; the types of healthcare coverage classified with national health insurance (NHI) and Medical Aids, which government guarantees the healthcare expenditures of low-income patients. In addition, the characteristics of chronic disease were considered with the types of disease and multiple chronic conditions. The types of chronic disease were categorized as hypertension, diabetes, or both, and multiple chronic conditions were defined as whether patients have chronic diseases other than hypertension and diabetes.

### 2.4. Statistical Analysis

To identify general characteristics of patients with chronic disease, frequency and descriptive statistics were analyzed. Furthermore, t-tests and ANOVA were conducted to determine the differences in annual average healthcare expenditures among patients. Finally, regression analysis, using a generalized estimation equation (GEE), was used to confirm the effects of age and duration of disease on annual healthcare expenditures, and the moderating effect of USOC.

GEE is a method that enables a longitudinal study design with data which is inappropriate for a normal distribution. Furthermore, it is an extension of a generalized linear model (GLM) that enables regression analysis for dependent variables, which have non-normal distributions such as binomial, Poisson, gamma, or multinomial [14].

The dependent variable of this study is healthcare expenditure, which is suitable for the gamma distribution model with a log-linked function. This model has been proven to be the most appropriate among various multivariable regression models for analyzing cost data, such as patients’ medical costs, which are usually skewed to the right [15]. Previous studies have also used GEE for longitudinal study designs to identify medical utilization and medical costs for patients who have asthma or diabetes with anxiety disorder [16,17]. Consequently, this study selected GEE as a method to estimate gamma regression with a log-linked function. Additionally, the Baron and Kenny model was used to confirm the moderating effects. The effect of only age and duration of chronic diseases on annual healthcare costs is presented in Model 1, and the moderating effects of USOC are specified in Model 2 with interactions between independent and moderating variables.

## 3. Results

In descriptive analyses, the frequencies and proportion of each categorical variables are reported, and the differences between each average of healthcare costs are presented in Table 1. For type of disease that was the primary focus of outpatient visits, patients with hypertension only, had the highest frequency (65.97%), followed by having both hypertension and diabetes (23.36%), and diabetes only (10.67%). In addition, 86.56% of patients had multiple chronic conditions excluding hypertension and diabetes. The most common type of visiting medical institution was in the order of clinic (75.49%), general hospital (14.11%), public health center (5.67%), and hospital (4.73%). The highest frequencies of general characteristics were as follows: 55.03% were female, 75.42% were married people, 25.79% were the lowest quintile of household income, 88.18% were disabled, and 95.48% had NHI. Finally, 50.98% of patients had a regular site for healthcare and 35.72% of patients had a regular doctor in 2012.

Table 1 also presents the average differences in annual healthcare expenditures according to the general characteristics of chronic disease patients. Firstly, the average of total healthcare costs differed by sex and disability. Furthermore, there were differences in both total healthcare and CDM costs depending on type of disease, multiple chronic conditions, medical institutions, and health coverage. According to the results, the patients who had both hypertension and diabetes had the highest CDM and total healthcare costs. Additionally, patients who had multiple chronic diseases along with hypertension and diabetes were found to spend an average of 65,494 and 258,431 won more on CDM and total healthcare costs. The type of visiting medical institutions were higher in the order of general hospitals, hospitals, clinics, and public health center in both CDM and total healthcare costs. Furthermore, compared to Medical Aid, NHI costs 172,150 and 332,672 won more in CDM and total healthcare. On the other hand, having a regular site or doctor differed only in CDM costs. Patients who had a regular site spent an average of 30,695 won more and who with a regular doctor spent 18,090 won more in CDM costs.

Table 2 presents the range and mean of the main variables used in this study. Patients’ age ranged from 30 to 100, with an average of 65.19 years. The average duration of disease was 7.63 years for hypertension, diabetes, or both, and ranged from 1 year to a maximum of 40 years. In addition, the annual average of CDM costs was 206,730 won with medical service costs (20.97%) and prescription drug costs (79.35%). The total healthcare costs were 455,382 won per year, with medical service costs (47.59%) and prescription drug costs (52.41%).

The effect of the general characteristics of patients with chronic diseases are presented in Table 3 and Table 4. For total amount of healthcare and CDM costs, it was confirmed that demographic characteristics such as marital status, household income, and disability did not affect changes in each type of healthcare cost. On the other hand, sex and marital status partially affected medical service costs, while prescription drug costs were affected by household income and disability. Furthermore, total healthcare and CDM costs increased when patients had diabetes or having both rather than hypertension only. In addition, when patients had multiple chronic conditions, total healthcare and CDM costs increased, while it decreased when they received Medical Aid. Finally, decreases in both CDM and total healthcare costs corresponded with use of smaller medical institutions.

In Table 3, the effects of patients’ age and duration of disease on total healthcare expenditure are presented, and Table 4 confirms the effects on CDM cost. Additionally, both tables indicate the moderating effects of USOC, such as of having a regular site or doctor. As patients’ age increased, total healthcare cost was increased (B = 0.005, *p* < 0.01; model 1). However, the CDM cost decreased with increasing age (B = −0.004, *p* < 0.05; model 1). On the other hand, increases in the duration of having hypertension or diabetes resulted in increasing total healthcare costs (B = 0.008, *p* < 0.05; model 1), and also increasing CDM costs (B = 0.008, *p* < 0.01; model 1). In addition, having a regular site had an impact on decreasing total healthcare cost (B = −0.085, *p* < 0.05; model 2), while it did not have a significant effect on CDM costs. Also, it was confirmed that both total healthcare (B = 0.078, *p* < 0.05; model 2) and CDM costs (B = 0.054, *p* < 0.05; model 2) increased when chronic patients had a regular doctor.

Finally, the moderating effect of having USOC was confirmed as follows. Firstly, when older patients with chronic diseases had a regular site, their CDM cost increased (B = 0.006, *p* < 0.05; Table 4), while it had no significant effect on total healthcare costs. Secondly, having a regular doctor by elderly patients did not significantly affect either total healthcare or CDM costs. Lastly, it was confirmed that if patients with longer duration had a regular site, both total healthcare (B = −0.018, *p* < 0.01; Table 3) and CDM costs (B = −0.014, *p* < 0.01; Table 4) decreased, while having a regular doctor by longer duration patients decreased CDM cost (B = −0.010, *p* < 0.05; Table 4).

## 4. Discussion

The purpose of this study was to highlight the importance of chronic diseases management and suggest appropriate directions for prevention and management plans by focusing on the differences in patient’s characteristics. For that reason, the study examined the moderating role of USOC on the association of annual healthcare expenditures with patients’ age and duration of chronic diseases. As a result, the effects of USOC were dissimilar depending on differences between aging and increases in diseases durations, and revealed consequences that could be an evidence to suggest proper preventive management plans for specific subjects.

According to the results, patients with a longer duration of hypertension or diabetes had higher CDM and total healthcare costs over time, along with more spending on prescription drugs. On the other hand, with the aging of patients, CDM costs reduced with decreases of medical service costs although the total amount of healthcare cost increased. The result indicates that elderly people with hypertension or diabetes need more focus on consistent management of their chronic diseases, which might reduce annual total healthcare costs by preventing complications. This is supported by prior studies that interventions for health promotion, such as population health management or disease management programs, are necessary for elderly patients with chronic conditions, and it might be the most cost-effective measure to lower healthcare costs by reducing the risk of disease and slowing disease progression [18,19].

For that reason, this study confirmed that having a regular site for patients with hypertension or diabetes decreased total healthcare costs, while having a regular doctor increased both CDM and total healthcare costs. It might be interpreted as having a regular site for chronic disease patients indirectly reduced annual total healthcare costs by health promotion and preventive management for chronic diseases even though it did not significantly affect CDM costs. Additionally, one study investigated that having a regular site in South Korea decreases outpatient visits and healthcare expenditures of hypertensive patients because it reduces inefficient outpatient utilization to general hospitals [20]. On the other hand, increases in CDM and total healthcare costs by having a regular doctor were accompanied by higher prescription drug costs. It seems that short-term increases in medication cost might be occurred because of specific medication counseling and management by a physician. This is supported by previous studies that having a regular doctor is more effective than having a regular site for preventive services, and stable physician-patient relationship might improve timely receipt of clinical prevention [21,22].

This study also confirmed a moderating effect of having a regular site. As patients became older without having a regular site, the cost for management of chronic diseases decreased. However, the moderating effect of having a regular site increased CDM cost for elderly, which might be optimistically interpreted as short-term increases because of periodic visits for prevention and management. This result is supported by a study that having a regular site for primary care increased more OOP costs than not having USOC [23]. In addition, even though total healthcare costs of elderly was not significantly affected by having a regular site, it represented a decreasing direction, indicated by a negative coefficients. This result can be interpreted as meaningful in the aspect of health promotion because total healthcare costs, which partially include CDM cost, were not increased even when CDM cost increased. In other words, having a regular site lowered total healthcare costs despite increase of age makes it higher. These results reflect other studies that preventive services by having USOC increases outpatient visits and healthcare expenditures but also it reduces inpatient spending in a long-term [24,25,26].

On the other hand, for patients with longer durations of hypertension or diabetes, having a regular site had an effect of reducing both CDM and total healthcare costs with decreases in medical service costs. Having a regular site for health management is strongly associated with receiving more medication treatments for chronic diseases [27]. However, this study presents contrary result that having a regular site reduces healthcare expenditures with less spending on medical services. It might represent that inefficient outpatient utilization decreased with proper management of chronic diseases. Additionally, it can be verified by a prior study that the number of outpatient visits for patients who have regular site was higher than national average of total outpatient visits in South Korea, and it could be reduced by decreases in secondary or tertiary healthcare [20].

Lastly, having a regular doctor had no significant effect on the annual healthcare expenditures of elderly patients with hypertension or diabetes, but it increased CDM cost for patients with a longer durations. These results indicate that specific medication and counseling by a regular doctor has more significant effect for patients who have longer duration of hypertension or diabetes than for elderly patients in health promotion by chronic diseases management.

Consequently, the result of this study was interpreted as suggestions of previous studies, which have to reduce healthcare expenditures through reduction of unnecessary medical utilizations and increase expenses for preventive management to reduce overall healthcare expenditure in the long-term [27,28,29,30,31]. This study confirmed that cost for chronic diseases management of elderly should be increased by having a regular site. In addition, increases in CDM cost and reduction of long-term healthcare expenditure through having a regular doctor should be focused by increases in disease durations rather than patient aging. In order to do this, it is necessary to improve patient’s reasonable healthcare utilizations along with strengthening the capability of primary healthcare with patient-specific USOC. Furthermore, by establishing partnership among physicians, patients, and communities, the reliability of chronic disease management should be ensured and provide information about related healthcare system.

This study is meaningful in comparing changes in annual healthcare expenditures according to age and disease duration of chronic patients, and in suggesting the effect of having a USOC for health promotion through CDM. However, this study had several limitations. First, other measurements of treatment continuity, such as medication compliance or Continuity of Care (COC) index, were not considered because of limitations of the data. Second, the data did not have longer follow-up, which the USOC was only measured in 2012–2013. Therefore, future research will be required to reflect a longer term of perspectives and various types of treatment continuity.

## 5. Conclusions

For patients who have hypertension or diabetes, having USOC is important to increase healthcare expenditures with purpose of preventive management, and to reduce long-term healthcare expenditures through health promotion. Therefore, reduction of chronic disease management of elderly people is required to be increased in a short term through constant visiting by having a regular site rather than having a regular doctor. On the other hand, patients with longer duration of hypertension or diabetes are necessary to increase their healthcare expenditures with continuous spending on preventive management by having a regular doctor. From the long-term perspective, as duration of chronic diseases increases, patients should have a regular site to improve their health status through steady follow-up for reducing total healthcare expenditures and to decrease inefficient healthcare utilizations.

## Figures and Tables

**Table 1:** Characteristics of study population and average differences in annual healthcare costs, 2012 (N = 1481).

Characteristics		N	%	CDM Costs		Total Healthcare Costs	
				Mean	t/F	Mean	t/F
Sex	Male	666	44.97	209,920	0.70	422,825	−2.62 **
	Female	815	55.03	204,124		481,987	
Marital status	Single	364	24.58	195,013	−1.63	446,650	−0.44
	Married	1117	75.42	210,549		458,227	
Household income	First quintile	382	25.79	188,747		462,764	
	Second quintile	330	22.28	208,466		480,577	
	Third quintile	301	20.32	218,232	1.83	442,698	0.60
	Fourth quintile	241	16.27	211,193		450,378	
	Fifth quintile	227	15.33	214,481		428,461	
Disability	No	1306	88.18	205,361	−0.91	447,210	−1.98 *
	Yes	175	11.82	216,954		516,366	
Type of disease	Hypertension	977	65.97	161,902		403,468	
	Diabetes	158	10.67	248,576	150.01 ***	484,161	24.52 ***
	Both	346	23.36	314,205		588,826	
Multiple chronic condition	No	199	13.44	150,036	−5.49 ***	231,676	−7.99 ***
	Yes	1282	86.56	215,530		490,107	
Medical institutions	General Hospital	209	14.11	360,527	102.18 ***	696,898	30.54 ***
	Hospital	70	4.73	253,330		510,953	
	Clinic	1118	75.49	180,932		419,400	
	Public health center	84	5.67	128,607		287,051	
Health coverage	NHI	1414	95.48	214,518	8.94 ***	470,432	6.22 ***
	Medical Aid	67	4.52	42,368		137,760	
Having a regular site	No	726	49.02	191,082	−3.75 ***	451,325	−0.35
	Yes	755	50.98	221,777		459,283	
Having a regular doctor	No	952	67.28	200,269	−2.11*	452,220	−0.38
	Yes	529	35.72	218,359		461,071	

Note: * *p* < 0.05, ** *p* < 0.01, *** *p* < 0.001.

**Table 2:** Summarization of patient’s age, duration of diseases, and annual healthcare costs, 2012 (N = 1481).

	Mean (%)	Standard Deviation	Minimum	Maximum
Age	65.19	11.11	30	100
Duration	7.63	5.93	1	40
CDM cost				
Medical service	43,344 (20.97)	62,787	750	818,300
Prescription drug	163,387 (79.35)	125,708	400	1,102,400
Total	206,730 (100)	158,027	1500	1,330,026
Total healthcare cost				
Medical service	216,728 (47.59)	344,142	1500	5,534,629
Prescription drug	238,654 (52.41)	179,341	500	1,452,240
Total	455,382 (100)	433,337	4500	5,862,019

**Table 3:** The results of generalized estimation equation (GEE) analysis for total healthcare cost.

Characteristics	Total Healthcare Cost											
	Model 1						Model 2					
	Medical Services		Prescription Drugs		Total Costs		Medical Services		Prescription Drugs		Total Costs	
	B	SE	B	SE	B	SE	B	SE	B	SE	B	SE
**Sex (ref: Male)**												
Female	0.212 **	0.060	0.024	0.035	0.108 **	0.038	0.216 ***	0.059	0.023	0.034	0.107 **	0.037
**Marital status (ref: Single)**												
Married	0.155	0.079	0.049	0.038	0.081	0.048	0.154 *	0.078	0.047	0.038	0.078	0.048
**Household income (ref: 1st quintile)**												
2nd quintile	0.028	0.103	0.005	0.028	0.024	0.063	0.020	0.099	0.006	0.028	0.022	0.061
3rd quintile	0.002	0.090	0.007	0.035	0.006	0.055	0.003	0.089	0.008	0.035	0.006	0.054
4th quintile	0.030	0.099	−0.015	0.035	−0.008	0.059	0.028	0.100	−0.016	0.035	−0.009	0.058
5th quintile	0.131	0.105	−0.009	0.038	0.033	0.064	0.126	0.102	−0.008	0.038	0.033	0.063
**Disability (ref: No)**												
Yes	0.086	0.090	0.134 *	0.055	0.100	0.059	0.103	0.088	0.136 *	0.055	0.108	0.058
**Type of disease (ref: Hypertension)**												
Diabetes	0.148	0.077	0.232 ***	0.057	0.178 **	0.056	0.165 *	0.075	0.232 ***	0.056	0.187 **	0.054
Both	0.043	0.072	0.412 ***	0.035	0.259 ***	0.042	0.049	0.071	0.416 ***	0.035	0.265 ***	0.041
**Multiple chronic condition (ref: No)**												
Yes	0.744 ***	0.094	0.292 ***	0.048	0.528 ***	0.056	0.723 ***	0.094	0.288 ***	0.048	0.515 ***	0.056
**Medical institution (ref: General Hospital)**												
Hospital	−0.243	0.125	−0.171 *	0.082	−0.244 **	0.085	−0.231	0.125	−0.163 *	0.082	−0.231 **	0.083
Clinic	−0.389 ***	0.070	−0.334 ***	0.043	−0.402 ***	0.045	−0.396 ***	0.070	−0.331 ***	0.043	−0.400 ***	0.044
Public health center	−0.733 ***	0.157	−0.607 ***	0.091	−0.718 ***	0.093	−0.724 ***	0.157	−0.600 ***	0.091	−0.705 ***	0.093
**Health coverage (ref: NHI)**												
Medical Aid	−1.117 ***	.154	−1.678 ***	.149	−1.429 ***	.136	−1.100 ***	.156	−1.680 ***	.149	−1.423 ***	0.137
**Age**	0.007 *	0.003	0.006 ***	0.002	0.005 **	0.002	0.011 *	0.005	0.007 ***	0.002	0.008 **	0.003
**Duration of Disease**	0.005	0.005	0.010 ***	0.003	0.008 *	0.003	0.017	0.010	0.013 ***	0.003	0.016 **	0.006
**Having a regular site (ref: No)**												
Yes							−0.161 **	0.061	−0.028	0.021	−0.086 *	0.035
**Having a regular doctor (ref: No)**												
Yes							0.109	0.059	0.046 *	0.022	0.078 *	0.034
**Interaction term**												
Age x regular site							−0.001	0.006	0.000	0.002	−0.001	0.003
Age x regular doctor							−0.008	0.005	−0.003	0.002	−0.005	0.003
Duration x regular site							−0.026 *	0.011	−0.005	0.003	−0.018 **	0.006
Duration x regular doctor							0.012	0.009	0.003	0.003	0.008	0.005

Note: * *p* < 0.05, ** *p* < 0.01, *** *p* < 0.001.

**Table 4:** The results of generalized estimation equation (GEE) analysis for CDM cost.

Characteristics	Total Healthcare Cost											
	Model 1						Model 2					
	Medical Services		Prescription Drugs		Total Costs		Medical Services		Prescription Drugs		Total Costs	
	B	SE	B	SE	B	SE	B	SE	B	SE	B	SE
**Sex (ref: Male)**												
Female	0.144 **	0.045	0.009	0.035	0.030	0.031	0.143 **	0.044	0.010	0.035	0.030	0.030
**Marital status (ref: Single)**												
Married	−0.009	0.055	−0.018	0.042	−0.022	0.036	−0.015	0.055	−0.017	0.042	−0.021	0.036
**Household income (ref: 1st quintile)**												
2nd quintile	0.061	0.050	0.049	0.037	0.053	0.032	0.067	0.049	0.052	0.037	0.055	0.033
3rd quintile	0.011	0.056	0.084	0.044	0.062	0.038	0.015	0.055	0.087 *	0.044	0.065	0.038
4th quintile	0.086	0.067	0.048	0.043	0.049	0.040	0.090	0.066	0.050	0.043	0.050	0.039
5th quintile	0.156 *	0.064	0.044	0.049	0.059	0.043	0.163 *	0.063	0.046	0.048	0.061	0.043
**Disability (ref: No)**												
Yes	−0.053	0.061	0.092	0.063	0.068	0.051	−0.058	0.061	0.092	0.063	0.069	0.051
**Type of disease (ref: Hypertension)**												
Diabetes	0.463 ***	0.074	0.199 **	0.065	0.258 ***	0.055	0.460 ***	0.072	0.205 **	0.065	0.263 ***	0.055
Both	0.523 ***	0.049	0.586 ***	0.044	0.577 ***	0.036	0.519 ***	0.049	0.590 ***	0.043	0.579 ***	0.035
**Multiple chronic condition (ref: No)**												
Yes	0.205 ***	0.050	0.114 *	0.045	0.135 **	0.040	0.201 ***	0.049	0.110 *	0.046	0.131 **	0.040
**Medical institution (ref: General Hospital)**												
Hospital	−0.612 ***	0.104	−0.170	0.096	−0.296 ***	0.081	−0.614 ***	0.103	−0.164	0.094	−0.290 ***	0.079
Clinic	−1.008 ***	0.062	−0.331 ***	0.050	−0.483 ***	0.045	−1.008 ***	0.061	−0.326 ***	0.050	−0.478 ***	0.045
Public health center	−2.151 ***	0.153	−0.544 ***	0.090	−0.795 ***	0.080	−2.136 ***	0.149	−0.527 ***	0.092	−0.779 ***	0.081
**Health coverage (ref: NHI)**												
Medical Aid	−1.108 ***	0.104	−2.153 ***	0.213	−1.821 ***	0.148	−1.101 ***	0.104	−2.165 ***	0.211	−1.827 ***	0.146
**Age**	−0.017 ***	0.002	0.000	0.002	−0.004 *	0.001	−0.018 ***	0.003	−0.001	0.002	−0.005 **	0.002
**Duration of Disease**	0.002	0.004	0.008 **	0.003	.008 **	0.003	.007	0.005	0.013 **	0.004	0.014 ***	0.003
**Having a regular site (ref: No)**												
Yes							0.044	0.044	−0.023	0.027	−0.013	0.024
**Having a regular doctor (ref: No)**												
Yes							0.032	0.045	0.067 *	0.028	0.054 *	0.025
**Interaction term**												
Age x regular site							0.003	0.004	0.005	0.003	0.006 *	0.002
Age x regular doctor							−0.001	0.004	−0.005	0.003	−0.004	0.003
Duration x regular site							−0.016 *	0.008	−0.012 *	0.005	−0.014 **	0.004
Duration x regular doctor							0.015	0.008	0.009	0.005	0.010 *	0.004

Note: * *p* < 0.05, ** *p* < 0.01, *** *p* < 0.001.
